# Responses of terrestrial ecosystem productivity and community structure to intra-annual precipitation patterns: A meta-analysis

**DOI:** 10.3389/fpls.2022.1088202

**Published:** 2023-01-09

**Authors:** Mingyu Xie, Lei Li, Bo Liu, Yalan Liu, Qian Wan

**Affiliations:** ^1^ State Key Laboratory of Desert and Oasis Ecology, Xinjiang Institute of Ecology and Geography, Chinese Academy of Sciences, Urumqi, China; ^2^ Xinjiang Key Laboratory of Desert Plant Roots Ecology and Vegetation Restoration, Xinjiang Institute of Ecology and Geography, Chinese Academy of Sciences, Urumqi, China; ^3^ Cele National Station of Observation and Research for Desert-Grassland Ecosystems, Cele, China; ^4^ University of Chinese Academy of Sciences, Beijing, China; ^5^ Shandong Provincial Key Laboratory of Soil Conservation and Environmental Protection, College of Resources and Environment, Linyi University, Linyi, China

**Keywords:** community structure, plant productivity, precipitation pattern, soil moisture, synthesis

## Abstract

**Introduction:**

The productivity and community structures of terrestrial ecosystems are regulated by total precipitation amount and intra-annual precipitation patterns, which have been altered by climate change. The timing and sizes of precipitation events are the two key factors of intra-annual precipitation patterns and potentially drive ecosystem function by influencing soil moisture. However, the generalizable patterns of how intra-annual precipitation patterns affect the productivity and community structures of ecosystems remain unclear.

**Methods:**

We synthesized 633 observations from 17 studies and conducted a global meta-analysis to investigate the influences of intra-annual precipitation patterns on the productivity and community structures of terrestrial ecosystems. By classifying intra-annual precipitation patterns, we also assess the importance of the magnitude and timing of precipitation events on plant productivity.

**Results:**

Our results showed that the intra-annual precipitation patterns decreased diversity by 6.3% but increased belowground net primary productivity, richness, and relative abundance by 16.8%, 10.5%, and 45.0%, respectively. Notably, we found that the timing uniformity of precipitation events was more important for plant productivity, while the plant community structure benefited from the increased precipitation variability. In addition, the relationship between plant productivity and community structure and soil moisture dynamic response was more consistent with the nonlinear model.

**Comclusions:**

The patterns of the responses of plant productivity and community structure to altered intra-annual precipitation patterns were revealed, and the importance of the timing uniformity of precipitation events to the functioning of production systems was highlighted, which is essential to enhancing understanding of the structures and functions of ecosystems subjected to altered precipitation patterns and predicting their changes.

## Introduction

Precipitation is a key factor driving the structures and functions of terrestrial ecosystems by affecting plant growth (Jongen et al., 2013; Zhang et al., 2018), plant productivity (Hu et al., 2010; Reichstein et al., 2013; Hu et al., 2018), plant community structure (Koerner et al., 2013; Jones et al., 2016), litter decomposition (Cui et al., 2021), and microbial community structure (Ochoa-Hueso et al., 2020). Global warming caused by human activities not only has altered total precipitation amount (Baker & Fritz, 2015) but also has increased the inter-annual and intra-seasonal variability in precipitation, resulting in frequent precipitation events and extreme events on the global, regional, and local scales (Trenberth et al., 2003; Sala et al., 2015; IPCC, 2022). Continuous extreme precipitation events undoubtedly alter the distribution of precipitation patterns and exert considerable impact on ecosystems by regulating plant productivity and community structure, eventually affecting global carbon cycles, even though precipitation amounts remain consistent (Sala et al., 2000; Knapp et al., 2002; Knapp et al., 2008). Hence, increasingly frequent changes in precipitation patterns are expected to regulate ecosystem processes to a greater extent than the other driving factors of global change (Fang et al., 2001).

Terrestrial ecosystems are highly sensitive to altered intra-annual precipitation patterns (Fay et al., 2003; Heisler-White et al., 2008; Didiano et al., 2016; Smith et al., 2016). Precipitation patterns influence ecosystem structure and function by affecting ecosystem water availability (Huang et al., 2010; Schwinning et al., 2003; Scott & Biederman, 2017). For example, the alterations to precipitation patterns can affect rainfall use efficiency by changing the stem leaf ratio and ultimately impact plant productivity (Yang et al., 2020). Altered precipitation patterns lead to the dominance of resource-conservative species with large root-shoot ratio and small specific leaf area (Fay et al., 2011). In general, intra-annual precipitation patterns are normally caused by alterations to the timing and sizes of precipitation events. In a semiarid steppe, increase in the size of precipitation events increases the amount of runoff and decreases evaporation losses (Robertson et al., 2009), and increase in interval between precipitation events increases soil moisture variability (Knapp et al., 2002) and reduces plant productivity (Fay et al., 2003), leaf carbon assimilation (Nippert et al., 2009), and soil CO_2_ outflow (Harper et al., 2005). At present, the direction and extent of terrestrial ecosystems worldwide response to altered precipitation patterns are still unclear due to the highly uncertainty of precipitation patterns.

Soil water availability directly affects plant growth and is commonly related to precipitation timing and size (Wang et al., 2016). First, large precipitation events alter the distribution ratio of soil moisture among different layers (Goldstein & Suding, 2014), thereby affecting the growth of plants using shallow or deep soil moisture. Second, increase in interval between precipitation events reduces ecosystem water use efficiency by extending the drying time of soil while decreasing the plant leaf area index (Liu et al., 2017) and stimulating respiratory pulses (Huxman et al., 2004). In addition, soil moisture changes caused by different distribution of precipitation events can also lead to significant differences in soil nutrients and nutrient use availability (Nitschke et al., 2017; Wang et al., 2017). For example, small precipitation events with short intervals can contribute to enhanced plant productivity by improving soil nutrient availability (Harpole et al., 2007; Nielsen & Ball, 2015). However, excessive precipitation will cause nutrient loss through leaching or surface flow, which is not conducive to plant growth (Yahdjian & Sala, 2010).Given that soil moisture is a link between precipitation and vegetation response (Wang et al., 2018), its variability limits plant transpiration and photosynthesis and affects ecosystem stability (Schneider et al., 2011). Therefore, assessing how the size and timing of precipitation events affect global terrestrial ecosystem productivity through soil moisture and their relative importance for plant productivity is necessary.

Ecosystems respond to altered intra-annual precipitation patterns, and the response depend on ecosystems type (Knapp et al., 2002; Zhang et al., 2013). For example, in three grassland ecosystems, tallgrass prairie showed 18% reduction in aboveground net primary productivity, whereas semiarid steppe and mixed prairie showed 30% and 70% increases when precipitation timing was altered (Heisler-White et al., 2009). In addition, precipitation variability plays a substantial role in ecosystems during the growing season (Bai et al., 2004; Swemmer et al., 2007; Robinson et al., 2013). For instance, a 24 year study in the Inner Mongolia grassland showed that the cumulative precipitation from January to July had a greater impact on plant productivity than precipitation in other periods (Bai et al., 2004). However, another study indicated that increased summer rainfall in combination with winter drought significantly increased diversity, and increased winter precipitation led to the emergence of new grass species conducted in semiarid steppe (Prevéy et al., 2014). Given the multitudinous distribution of intra-annual precipitation patterns and ecosystem types, the effects of precipitation patterns on terrestrial ecosystems on the global scale are inadequately studied, and thus further assessment of how terrestrial ecosystems respond to climate change is currently limited. Therefore, the responses of ecosystems and experimental periods to altered intra-annual precipitation patterns should be investigated on the global scale.

In response to the current research gap, we conducted a meta-analysis using 633 observations from global precipitation manipulation experiments to synthesize the effect of altered precipitation patterns on terrestrial ecosystem productivity and community structure. Using a comprehensive dataset, we analyzed the response of plant productivity and community structure to intra-annual precipitation patterns and how the size and timing of precipitation events affect plant productivity by altering soil moisture. We asked two questions: How will plant productivity and community structure respond to intra-annual precipitation patterns? How important are the size and timing of precipitation events to ecosystems and how do they affect plant productivity through soil moisture? Based on the above two research questions, we proposed the following hypothesis: (1) Altered intra-annual precipitation patterns will increase plant belowground productivity and reduce diversity. (2) The timing uniformity of precipitation events will reduce soil moisture variability, increase soil moisture content and contribute to plant productivity. Precipitation pattern affects plant productivity and community structure by changing soil moisture availability, and the relationship between them is more consistent with the nonlinear model.

## Materials and methods

### Data collection and extraction

We used the Web of Science database to search peer-reviewed publications (2000-2021.10) on primary production and community structure with the following keywords: (rainfall distribution OR precipitation distribution OR rainfall event* OR precipitation event* OR rainfall regime* OR precipitation regime* OR rainfall pattern* OR precipitation pattern* OR rainfall frequency OR precipitation frequency) AND (net primary product* OR community structure OR divers* OR species divers* OR species rich* OR composition shifts OR primary product* OR ANPP OR BNPP OR NPP) AND (experiment* OR treatment*). Studies were incorporated when they met the following criteria: (1) precipitation manipulated must be carried out in natural terrestrial ecosystems, excluding studies conducted in laboratories; (2) plant communities were not artificial cultivated; (3) ambient and treatment were performed under the same biotic and abiotic conditions; (4) the study included at least one productivity variables or community structure indicators, and the duration of the experiment was clearly reported; (5) for years of experimental observation results, only results reported separately by year were collected; (6) for the treatment group controlled by precipitation patterns, temperature, N addition, added root-feeding scarabs, grazed and other factors, the multi-factor treatment group, such as temperature and N addition, was used as the ambient group for meta-analysis. If experiments at multiple sites were reported in an article, they were treated as independent studies.

**Figure 1:**
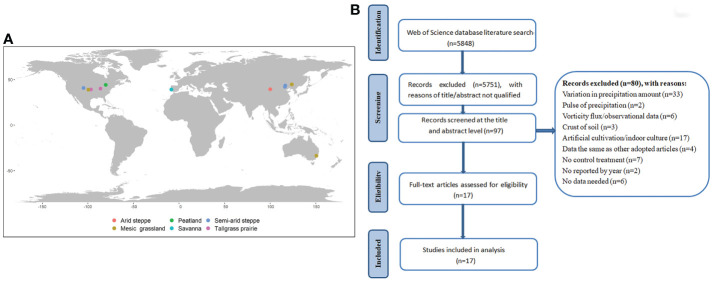
Map of the global distribution of study sites **(A)** and a flow chart of criteria for study selection **(B)** used in this meta-analysis. The distribution of 17 studies in 19 different biomes.

Using these criteria, we obtained 633 observations results from 17 published studies (**Figure 1B**; **Table S1**; **Supplementary File 1**). Base on precipitation frequency of experimental and ambient conditions, we divided them into two groups. In the different precipitation frequency group (the precipitation frequency of experimental and ambient were inconsistent), 207 observations reported even event timing (*e*_timing_, defined as the uniform precipitation event timing), 26 reported even event size and timing (even, defined as uniform precipitation event size and timing), and 229 reported non-even event size and timing (non-even, defined as non-uniform precipitation event size and timing). In same precipitation frequency group (the precipitation frequency of experimental and ambient were consistent), 11 had even event size (*E*_size_, defined as uniform precipitation event size), 12 had even event timing (*E*_timing_, defined as the uniform precipitation event timing), 11 had even event size and timing (EVEN, defined as uniform precipitation event size and timing), and 137 had non-even event size and timing (NON-EVEN, defined as non-uniform precipitation event size and timing). Soil nutrient data were only obtained from 3 studies, which mainly included NO_3_^-^ concentrations, NH_4_^+^ concentrations, total N, carbon pools, nitrogen pools and C:N. We failed to analyze the impact of altered precipitation pattern on plant productivity due to insufficient data.

We used GetData Graph Digitizer 2.26 to extract the means, sample sizes (if reported), and standard errors for each study. Data were derived from tables, figures, and appendices of the original publications. For each study, we collected information about latitude, longitude, mean annual temperature (MAT), and precipitation (MAP), altitude, ecosystem type, and experiment duration (i.e., the start and end dates of the experiment). For articles missing MAT and MAP, we used the names of countries, states, or experimental study sites to search for other publications. In addition, we also collected soil background data (including soil texture, pH, soil organic matter content, total N and total P) at the study sites, but most of the studies lacked relevant data (**Table S1**). Given that most of the ecosystem types we collected were grassland ecosystems, we classified grassland ecosystems by the precipitation and vegetation types of the research sites. The collected articles were grouped into different biomes: arid steppe, semi-arid steppe, mesic grassland, tallgrass prairie, peatland, and savanna (**Figure 1A**). Due to the few global precipitation pattern manipulation experiments, there is a lack of precipitation pattern manipulation experiments conducted in South America and Africa in the publications we collected.

### Variable selection

We selected plant productivity and community structure from published studies and divided them into four categories of response variables (**Table S2**): (1) biomass, usually refers to the total amount of live organic matter in per unit area at a certain time, including aboveground biomass (AGB) and belowground biomass (BGB); (2) net primary productivity, aboveground net primary productivity (ANPP) was calculated from the peak biomass per unit area aboveground with no carry of live biomass from previous years. Belowground net primary productivity (BNPP) was estimated according to the dry mass of root growth per unit area per unit time, which was measured by root growth into cores and soil drilling; (3) community structure indicators, species richness is simply the number of species per unit of area, and the usual measures are typically separated into measures of α, β, and γ diversity (Brown et al., 2007). Cover can be used in measuring the luxuriance and growth situation of vegetation. Shannon-Wiener (*H*) index reflects the diversity of plant community according to the number of species. Pielou’s evenness index (*E*) can reflect the evenness of plant community. Relative abundance refers to the abundance of one species as a percentage of the total abundance of all species in a community; (4) root-shoot ratio, refers to the ratio of fresh or dry weight between the belowground and aboveground parts of a plant and is used in assessing changes in carbon allocation in biomass or carbon allocation in response to climate change (Song et al., 2019).

### Data analysis

Effect size can be compared, and the treatment effects of all studies can be expressed on a common scale and used in highlighting general responses over a broad range of ecosystems (Wu et al., 2011). We used log response ratio (ln*RR*) to test the responses of plant productivity and community structure to intra-annual precipitation patterns following Hedges et al. (1999):


$$\text{ln}RR=\ln\left(\frac{Xt}{Xc}\right)=\ln\left(Xt\right)-\ln\left(Xc\right)$$

where *Xt* and *Xc* are the mean values of productivity and community structure, respectively, in the treatment and ambient groups. Variance in each ln*RR* was calculated as follows:


$$\nu =\frac{SD_{t}^{2}}{N_{t}X_{t}^{2}}+\frac{SD_{c}^{2}}{N_{c}X_{c}^{2}}$$

where *SD_t_* and *SD_c_* represent standard deviations; *N_t_* and *N_c_* represent sample sizes of the treatment and ambient groups, respectively.

We used MetaWin (Version 2.1, Rosenberg et al., 1997) to calculate the weighted response ratio (ln*RR*++) and 95% confidence interval (95% CI) of the mixed-effects. The effects of precipitation patterns were considered significant (*p* < 0.05) when 95% CI of response variables does not overlap with zero. To determine whether the responses were different among groups, we grouped the response variables according to the information collected (ecosystem type, precipitation distribution, and experiment period) and conducted a between-group heterogeneity test (*Q*_B_ tests). If *Q*_B_ values were significant (*p* < 0.05), the responses were considered significantly different among groups (Liu et al., 2016). We converted weighted response ratios (ln*RR*++) and there 95% CIs for each explanation back to the percentage change as follows: (e^ln^ *^RR^* ^++^ − 1) x 100%.

We examined differences in plant productivity and community structure among different treatments according to the research method of Griffin-Nolan et al. (2021). We divided the treatment groups according to precipitation pattern in the growing season: (1) non-even event size and timing (non-even/NON-EVEN), (2) even event size (*E*_size_), (3) even event timing (*e*_timing_/*E*_timing_), and (4) even event size and timing (even/EVEN). We used different soil moisture indicators to evaluate difference in soil moisture among the treatment groups. Soil moisture indicators included coefficient of variation (CV) of soil moisture, change in soil moisture (CSWC), median soil moisture, the consecutive disparity index (D) of soil moisture, soil moisture skewness, and soil moisture kurtosis. The CV is one of the most commonly used indicators for evaluating time variability (Fernández-Martínez et al., 2018), but they have mean dependence on or high sensitivity to rare events. By using the consecutive disparity index, we can evaluate the rates of changes in time for consecutive values. The calculation formula of consecutive disparity index is as follows:


$$D=\frac{1}{n-1}\sum_{i=1}^{n-1}|\ln\frac{p_{i+1}}{p_{i}}|$$

where *pi* is the series value at time *i* and *n* is the series length (Fernández-Martínez et al., 2018).

The TSA package in R V.4.0.2 (R Core Team, 2020) was used in calculating the skewness and kurtosis of soil moisture (Chan et al., 2020). We used SPSS 25.0 to analyze the soil moisture among treatments. Differences in plant productivity and community structure were analyzed by independent sample t-test and one-way analysis of variance (Duncan). Linear and polynomial regression models were used in determining the relationship between soil moisture and relative changes in predictor variable.

## Results

The observations we collected in compliance with the criteria were mainly distributed in North America and Asia (**Figure 1A**). The study areas were located from 33.61 S to 44.67 N, and the MAT of experimental sites ranged from -0.48°C to 17.0°C and MAP ranged from 117 mm to 835 mm. The treatment periods of these studies fall into the growing season (May-October) and non-growing season (November-May; **Table S1**).

### Overall of plant productivity and community structure response to intra-annual precipitation patterns

**Figure 2:**
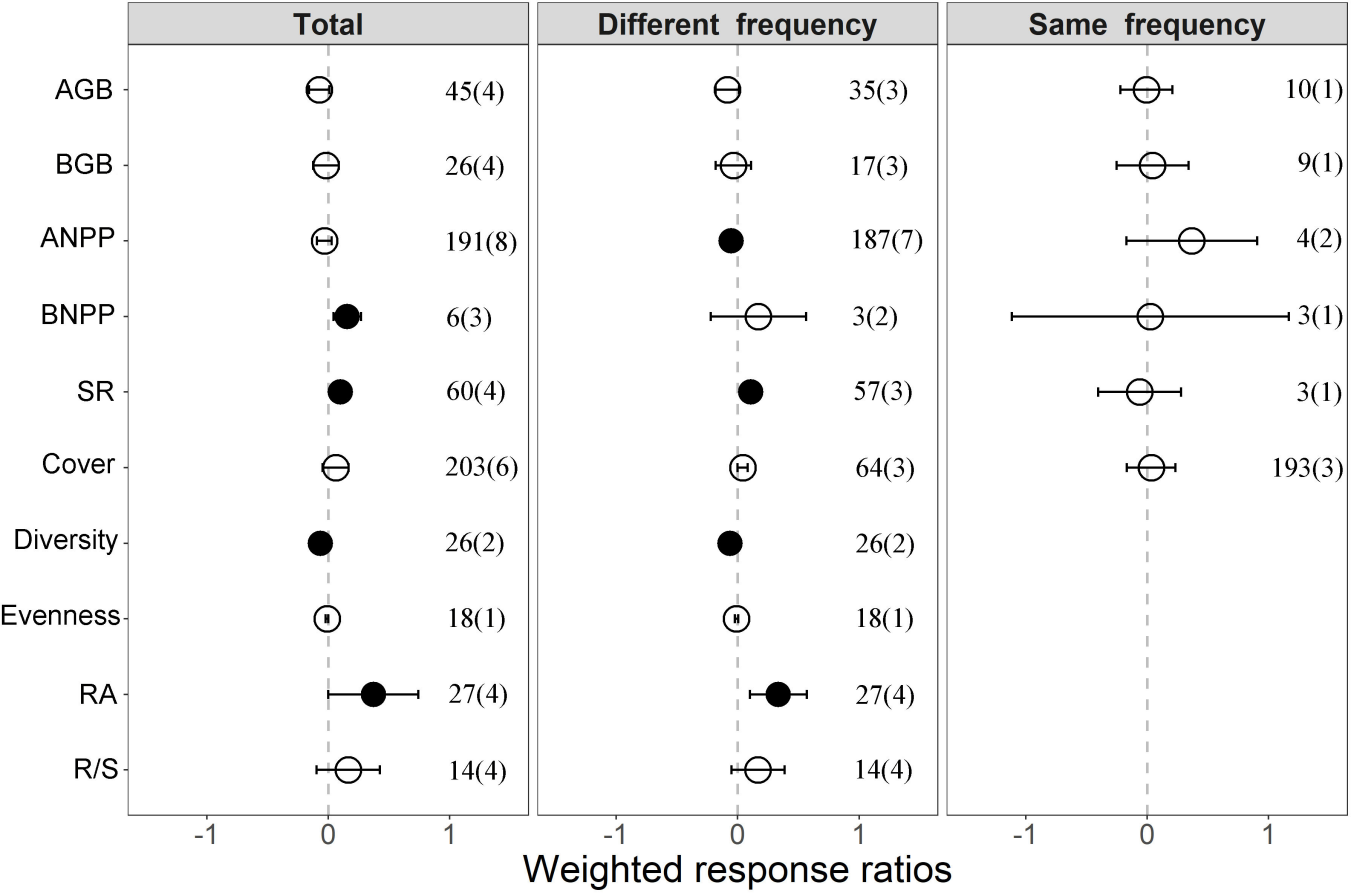
Responses of plant productivity and community structure to intra-annual precipitation patterns (including total precipitation pattern group, different precipitation frequency group and same precipitation frequency group). Values are weighted response ratios and their 95% confidence intervals (95% CI). Values indicate the intensity of the impact of altered intra-annual precipitation distribution on plant productivity and community structure relative to the values in the ambient treatment. The vertical dashed line represents weighted response ratios = 0. If 95% CI did not overlap with zero, the effects of precipitation pattern on variables were considered significant (denoted by black circles). Numbers corresponding to each variable represent the number of data observations, and the number in parentheses represents the number of studies. AGB, aboveground biomass; BGB, belowground biomass; ANPP, aboveground net primary productivity; BNPP, belowground net primary productivity; SR, species richness; RA, relative abundance; R/S, root–shoot ratio.

In general, altered intra-annual precipitation significantly affected plant community structure but had little effect on plant productivity (**Figure 2**). Compared with ambient treatment, altered intra-annual precipitation significantly enhanced BNPP, richness, and relative abundance by 16.8%, 10.5%, and 45.0%, respectively but decreased diversity by 6.3% in the total precipitation pattern group. In the different precipitation frequency group, ANPP and diversity were significantly reduced by 5.1% and 6.3%, whereas richness and relative abundance remained consistent with the total precipitation pattern, increasing by 11.2% and 39.7%, respectively. By contrast, all variables have no significant changed in the same precipitation frequency group.

**Figure 3:**
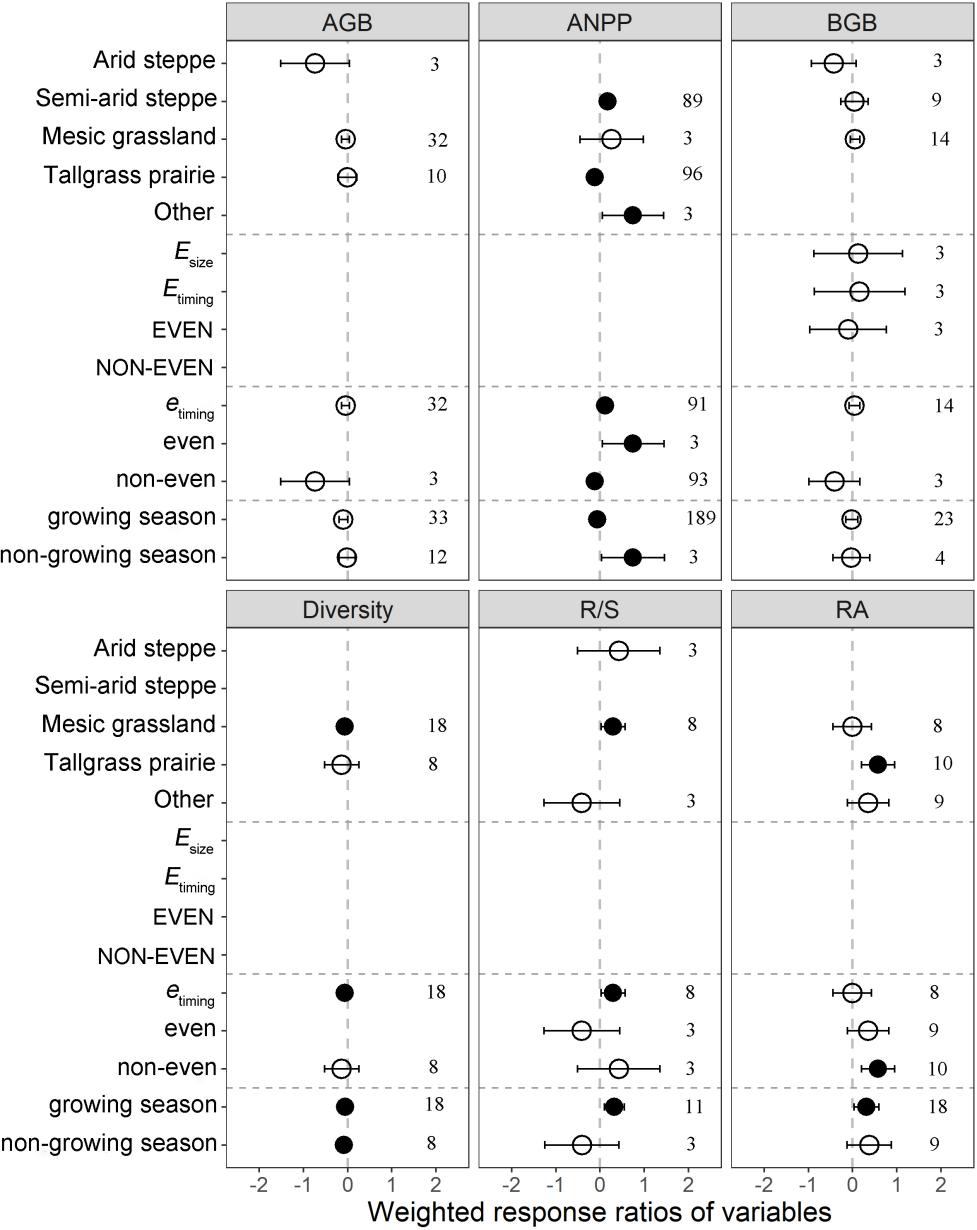
Subgroup analysis of plant productivity and community structure on altered intra-annual precipitation patterns. Between-group heterogeneity (*Q*_B_) was tested according to ecosystem type, precipitation distribution and experimental period. If 95% CI did not overlap with zero, the effects of precipitation pattern on variables were considered significant (denoted by black circles). Error bars represent 95% confidence intervals (CI). The vertical dashed line represents weighted response ratios = 0. Numbers indicate the number of data observations. The *Q*_B_ statistical test was used to compare the differences in weighted response ratios among groups divided by ecosystem type, precipitation distribution and experimental period. A significant *Q*_B_ value (*p* < 0.05) suggested that the weighted response ratios of a given variable differed among groups. *E*_szie_, even event size treatment; *E*_timing_, even event timing treatment; EVEN, even event size and timing treatment; NON-EVEN, non-even event size and timing treatment; *e*_timing_, even event timing treatment; even, even event size and timing treatment; non-even, non-even event size and timing treatment. See **Figure 2** for abbreviations.

Plant productivity exhibited different responses under the group of ecosystem type, precipitation distribution and experimental periods (**Figure 3**; **Table S3**). Significant difference in ANPP was observed among the ecosystem types with the changing of intra-precipitation patterns (*p* < 0.001).The responses of ANPP in semi-arid steppe (19.16%) and other ecosystem (110.85%) increased significantly, whereas the response in tallgrass prairie (-11.42%) decreased significantly (**Figure 3**). The responses of ANPP to altered intra-annual precipitation patterns showed significant difference among different precipitation frequency (*p* < 0.001). The ANPP values of the *e*_timing_ and even treatments increased by 12.54% and 110.90%, whereas the ANPP of the non-even treatment decreased by 11.15% (**Figure 3**). Alterations to precipitation patterns in the growing season reduced ANPP by 6.23%, whereas the ANPP in the non-growing season increased by 110.96% (**Figure 3**).

The effects of intra-annual precipitation patterns on plant community structure differed by ecosystem type, precipitation distribution, and experimental period (**Figure 3**; **Figure S1**; **Table S3**). Across ecosystems, the responses of root–shoot ratio increased by 34.38% only in the mesic grassland. Similarly, only *e*_timing_ treatment significantly enhanced root-shoot ratio by 34.38% at different precipitation frequency. In the growing season, significantly increase in root–shoot ratio by 38.20%, while no change was found in the non-growing season (**Figure 3**). In all groups, only difference precipitation frequency cover reached a statistically significant level (*p* < 0.001; **Figure S1**). Among them, *e*_timing_ treatment significantly reduced cover by 12.36%, while non-even treatment significantly increased cover by 7.42%. Across ecosystems, tallgrass prairie significantly increased richness by 11.43%, whereas semi-arid steppe and mesic grassland did not affect richness (**Figure S1**).

### Effects of intra-annual precipitation patterns on soil moisture

**Figure 4:**
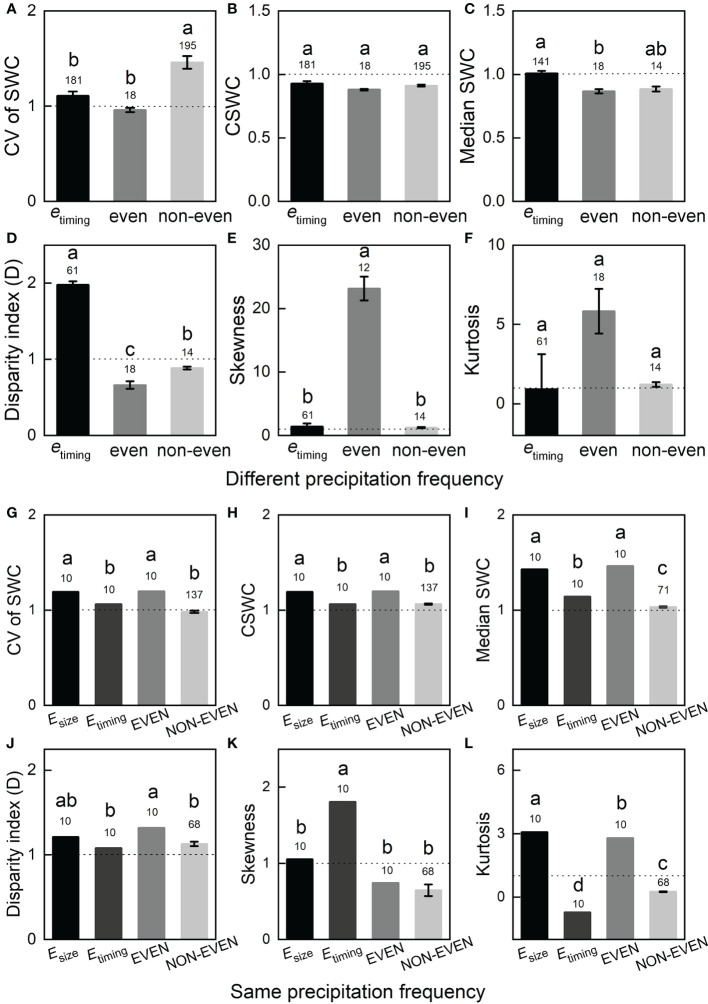
The bar chart indicate the treatment effects on multiple indices of soil moisture including **(A, G)** the coefficient of variation (CV) of soil moisture, **(B, H)** change in soil moisture (CSWC), **(C, I)** median soil moisture, **(D, J)** the consecutive disparity index (D) of soil moisture, **(E, K)** soil moisture skewness, **(F, L)** soil moisture kurtosis in different precipitation frequency and same precipitation frequency. Data are reported as mean ± SE, and numbers indicate the number of data observations. The dotted lines indicate the reference line for treatment intensity. Significant differences among treatments (*p* < 0.05) are indicated by different letters. See **Figure 3** for abbreviations.

In the different precipitation frequency groups, CV in soil moisture was lower in the even treatment, while was higher in the *e*_timing_ treatment and non-even treatment compare with the ambient treatment, and the non-even treatment was significantly higher than other two treatments (*p* < 0.001; **Figure 4A**). As for change in soil moisture, three treatments were lower than ambient treatment, and no significant difference was found among all treatments (*p* = 0.516; **Figure 4B**). The median soil moisture in the *e*_timing_ treatment was significantly higher than that in the even treatment (*p* < 0.01), and no significant difference was found among other treatments (**Figure 4C**). The consecutive disparity index (D) in the *e*_timing_ treatment was higher than that in the ambient treatment, with and significant differences were observed among all treatments (*p* < 0.001; **Figure 4D**). The even treatment had the highest skewness and kurtosis in soil moisture, but the even treatment had significantly higher soil moisture skewness than the other treatments (*p* < 0.001; **Figure 4E**). No significant difference in soil moisture kurtosis was found among all treatments (*p* = 0.435; **Figure 4F**).

In the same precipitation frequency group, the CV, change in soil moisture and median of soil moisture in the *E*_size_ and EVEN treatments were significantly higher than those in the *E*_timing_ and NON-EVEN treatments (**Figures 4G-I**). Moreover, the median soil moisture in the *E*_timing_ treatment was significantly higher than that in the NON-EVEN treatment (*p* < 0.001; **Figure 4I**). The EVEN treatment had significantly higher D than the *E*_timing_ treatment and NON-EVEN treatment (*p* < 0.05), and no significant difference was found among other treatments (**Figure 4J**). The soil moisture skewness in the *E*_timing_ treatment was significantly higher than that in the other treatments (*p* < 0.001; **Figure 4K**). Significant difference in soil moisture kurtosis was observed among all treatments (*p* < 0.001; **Figure 4L**).

### Plant productivity and community structure response to precipitation event size and timing

**Figure 5:**
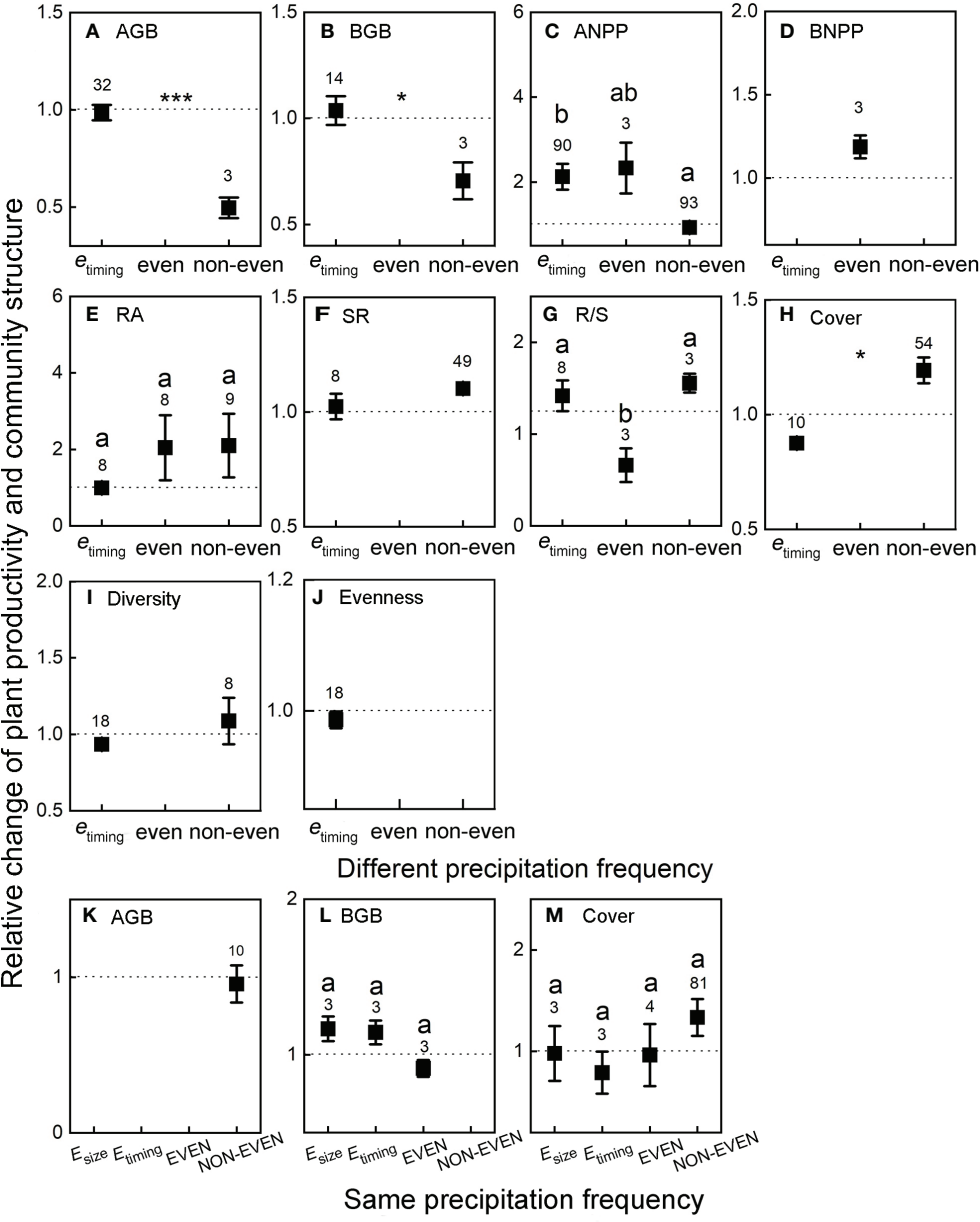
Treatment effects on plant productivity and community structure relative to the ambient in different precipitation frequency **(A–J)** and same precipitation frequency **(K–M)**. Data are reported as mean ± SE and numbers indicate the number of data observations. The dotted lines indicate the reference line for treatment intensity. Significant differences among treatments are indicated by different lettering (*p* < 0.05) and * (*p < 0.05, ***p < 0.001). See **Figure 2** and **Figure 3** for abbreviations.

In general, plant productivity and community structure were changed in different precipitation frequency (**Figure 5**). AGB and BGB in the non-even treatment were significantly lower than those in the *e*_timing_ treatment (*p* < 0.05; **Figures 5A, B**). In the difference precipitation frequency group, decrease in the time variation of precipitation resulted in an increase in ANPP in all treatments, and the order of the treatments by time variation was as follows: non-even < *e*_timing_ < even. In addition, ANPP was significantly lower in the non-even treatment than in the *e*_timing_ treatment (*p* < 0.001; **Figure 5C**). By contrast, the cover of non-even treatment was significantly higher than that in the *e*_timing_ treatment (*p* < 0.05; **Figure 5H**). The root-shoot ratios of the *e*_timing_ and non-even treatments were significantly higher than the root-shoot ratio of the even treatment (*p* < 0.05; **Figure 5G**).

The same precipitation frequency treatment had little effect on plant productivity and community structure (**Figure 5**). BGB in *E*_size_ and *E*_timing_ treatments were higher than that in ambient treatment and the EVEN treatment (**Figure 5L**). The cover only in NON-EVEN treatment was higher than that in the ambient treatment (**Figure 5M**). However, no significant differences in BGB and cover were found among all treatments (*p* = 0.081; *p* = 0.847; **Figures 5L, M**).

### Factors influencing the responses of plant productivity and community

In the total precipitation pattern group, BGB had significant relationship with the multiple indices of soil moisture, which had negatively correlated relationships among the BGB, consecutive disparity index, and kurtosis of soil moisture and was positively correlated with soil moisture skewness, promoting the fitness of BGB with the CV of soil moisture, median soil moisture, and soil moisture change after quadratic and quartic polynomial fitting (**Table 1**; **Figure S2**). ANPP increased as soil moisture changed, and had quartic polynomial relationship with the consecutive disparity index of soil water (**Table 1**; **Figure S2**). Moreover, the correlation among the diversity and evenness and median soil moisture was quadratic fitting (**Table 1**; **Figure S3**). The richness and soil moisture change were matched cubic fitting, and the root-shoot ratio was negatively correlated with soil moisture change (**Table 1**; **Figure S4**).

**Table 1:** Effects (R^2^ and *p*-values) of soil moisture indices on plant productivity and community structure.

Total precipitation pattern																	
Variable	CV of SWC		CSWC		Median SWC			Disparity index (D)				Skewness				Kurtosis	
	R^2^	*p*	R^2^	*p*	R^2^		*p*	R^2^	*p*			R^2^		*p*		R^2^	*p*
AGB	0.015	0.470	0.146	0.142	0.051	0.289		0.035	0.378		0.112		0.099			0.041	0.345
BGB	0.355	0.019*	0.555	0.008**	0.591	0.011*		0.602	0.002**		0.609		0.002**			0.417	0.017*
ANPP	0.016	0.099	0.039	0.009**	0.002	0.650		0.599	0.041*		0.072		0.332			0.070	0.339
Cover	0.000	0.964	0.003	0.508	0.001	0.790		0.008	0.504		0.002		0.723			0.002	0.717
Diversity	0.018	0.565	0.185	0.052	0.588	0.005**		0.007	0.772		0.021		0.608			0.102	0.246
Evenness	0.065	0.380	0.015	0.677	0.591	0.007**		0.500	0.022*		0.007		0.770			0.002	0.873
RA	0.014	0.550	0.045	0.290	0.157	0.292		0.193	0.236		0.125		0.350			0.016	0.747
SR	0.049	0.112	0.206	0.010**	NA	NA		NA	NA		NA		NA			NA	NA
R/S	0.259	0.110	0.831	0.000***	NA	NA		NA	NA		NA		NA			NA	NA
Different precipitation frequency																	
AGB	0.045	0.296	0.280	0.005**	0.057	0.338		0.139		0.128	0.134		0.136		0.106		0.188
BGB	0.541	0.030*	0.787	0.005**	NA	NA		NA		NA	NA		NA		NA		NA
ANPP	0.016	0.102	0.040	0.009**	0.003	0.584		0.070		0.407	0.072		0.332		0.067		0.416
Cover	0.018	0.324	0.169	0.002**	0.537	0.004**		0.643		0.000***	0.017		0.669		0.049		0.470
Diversity	0.018	0.565	0.185	0.052	0.588	0.005**		0.007		0.772	0.021		0.608		0.102		0.246
Evenness	0.065	0.380	0.015	0.677	0.591	0.007**		0.500		0.022*	0.007		0.770		0.002		0.873
RA	0.014	0.550	0.045	0.290	0.157	0.292		0.193		0.236	0.125		0.350		0.016		0.747
SR	0.041	0.159	0.204	0.001**	NA	NA		NA		NA	NA		NA		NA		NA
R/S	0.259	0.110	0.831	0.000***	NA	NA		NA		NA	NA		NA		NA		NA
Same precipitation frequency																	
AGB	0.029	0.639	0.000	0.961	0.092	0.559		0.092		0.559	0.092		0.559		0.092		0.559
BGB	0.128	0.344	0.128	0.344	0.153	0.298		0.348		0.095	0.253		0.167		0.079		0.465
Cover	0.005	0.521	0.000	0.898	0.005	0.619		0.003		0.703	0.017		0.385		0.004		0.679

The implications of soil moisture indices are defined in the legend to **Figure 4**.

p-values < 0.05 are considered significant.

*p < 0.05 **p < 0.001 ***p < 0.001.

In the different precipitation frequency group, soil moisture change, AGB, and ANPP were significantly positively correlated, whereas BGB showed a quadratic and cubic fitting relationship with the CVs of soil moisture and soil moisture change (**Table 1**; **Figure S5**). Increase in soil moisture change and median soil moisture increased cover, whereas increase in the soil moisture consecutive disparity index reduced cover (**Table 1**; **Figure S6**). In addition, median soil moisture had a quadratic fitting relationship with diversity and evenness (**Table 1**; **Figure S6**). We also found that the richness and root-shoot ratio were negatively correlated with soil moisture change **(** **Table 1**; **Figure S7**).

## Discussion

In this meta-analysis, we first investigated the responses of plant productivity and community structure to intra-annual precipitation patterns, and further compared differences in these factors among varied treatments at the same precipitation frequency and different precipitation frequency on the global scale. Our results suggested that different precipitation frequency reduced ANPP, while total precipitation frequency significantly enhanced BNPP. Both precipitation frequencies had positive effects on richness and relative abundance, and have a negative effect on diversity. Plant productivity and community structure showed higher levels of responses to ecosystem types and difference precipitation frequency. Moreover, ecosystem functioning was more influenced by the timing uniformity of precipitation events, whereas the combination of increased precipitation event size and timing variability had greater effects on plant community structure. More importantly, the relationship among soil moisture and plant productivity and community caused by altered precipitation pattern were more aligned with the nonlinear model.

### Plant productivity

The responses of ANPP and BNPP to the intra-annual precipitation patterns were different. In the total precipitation pattern group, the response of ANPP decreased slightly, but the difference was nonsignificant. Meanwhile, BNPP increased significantly (**Figure 2**). The response of ANPP decreased significantly, while BNPP did not change significantly in the difference precipitation frequency group (**Figure 2**). In addition, the response of productivity had no significant changes in the same precipitation frequency group. These results can be attributed to three reasons. First, the responses of ANPP and BNPP to soil moisture, which was regulated by precipitation frequency, may have been different. In the different precipitation frequency group, all of the collected articles showed increased precipitation event size and decreased frequency. Thus, low precipitation frequency and extended precipitation interval would reduce temporally soil moisture availability and increased duration of soil desiccation (Liu et al., 2017), thereby exacerbating drought stress during two precipitation events and was adverse to plant growth (Knapp et al., 2002). Second, altered precipitation patterns generally stimulated plant root growth resistance to soil water deficit to prevent even large reductions in aboveground productivity (Fay et al., 2003). These results were consistent with our results that the response of root-shoot ratio increased to intra-annual precipitation pattern, although no significant change was observed (**Figure 2**). Finally, altered intra-annual precipitation pattern affect ecosystem productivity, mediate the availability of soil nutrients, such as excessive precipitation, and would lead to nutrient loss because of leaching, thereby limiting plant growth and productivity (Yahdjian & Sala, 2010). Notably, the synthetic effects of soil nutrients on ecosystem productivity were deficient because of insufficient data.

Across ecosystems, altered intra-annual precipitation pattern significantly increased the ANPP in semiarid steppe and other ecosystems, but decreased the ANPP in tallgrass prairie (**Figure 3**). Although small rainfall events can intermittently alleviate water stress and improve plant water status (Yahdjian & Sala, 2010), high atmospheric evaporation demand rapidly depletes soil water after these small rainfall inputs (Scott and Biederman, 2017) due to soil moisture stress and inferior availability in semi-arid steppe (Peng et al., 2013). Therefore, large precipitation size enables water to access deep soil and increases the duration of high soil moisture pulse (Moore et al., 2020), and the amount of water consumed through transpiration increases; thus, plant productivity improves. As for tallgrass prairie, increased interval between precipitation events can reduce plant biomass because C_4_ grasses are strongly limited; while C_3_ grasses and forbs are resistant to altered precipitation variability (Fay et al., 2003). In the different precipitation frequency group, *e*_timing_ and even treatments significantly increased ANPP, whereas non-even treatment significantly decreased ANPP (**Figure 3**). Each treatment with progressively reduced precipitation variability allocated this precipitation evenly and thus increased and maintained soil moisture pulse in the growing season, eliminating the challenge imposed by seasonal precipitation environment on plants (Moore et al., 2020; Griffin-Nolan et al., 2021).

### Plant community structure

Our results showed that the responses of richness and relative abundance to precipitation pattern significantly increased and diversity decreased significantly on a global scale (**Figure 2**). Similarly, given that most of the collected literature has shown reduction in the frequencies of precipitation events. This reduction changed the resource allocation of water between shallow and deep soil may contribute to the growth of plants using deep soil water, while exacerbating the stress of plants using shallow soil water (Jones et al., 2016). Therefore, altered precipitation pattern decreased the abundance of one species and then would be compensated by increases in the abundance of other species, resulting in strong species asynchrony (Hallett et al., 2014). In addition, dominant plants usually can resist variable precipitation patterns (Fay et al., 2011). Hence, altered precipitation patterns reduced plant species evenness by affecting plant abundance, consistent with our results (**Figure 2**). Given that species diversity is a comprehensive reflection of species richness and evenness (Strong, 2016) which was significantly reduced by an altered precipitation pattern (**Figure 2**).

Responses of different ecosystems were distinct. Our results showed that the response of diversity in mesic grassland was reduced, and responses of relative abundance and richness in tallgrass prairie increased (**Figure 3**; **Figure S1**). These discrepancies can be explained by increased time interval between precipitation events, which reduces soil moisture in mesic grasslands (Heisler-White et al., 2009). Reduction in soil moisture in turn reduces the number of plant species because plants have low resistance to high precipitation variability. By contrast, tallgrass prairie can resist long-term precipitation variability and is relatively resilient to short-term extreme precipitation, and plants can utilize deep soil moisture (Jones et al., 2016).

As individual ecosystems typically provide few observations with little statistical power (Button et al., 2013), we tested their overall effect. It was found that the precipitation distribution pattern with decreasing precipitation frequency and increasing precipitation interval may significantly affect plant productivity and community diversity by changing the soil water available to plants and its availability, and this precipitation distribution pattern has a significant effect on plant growth in semi-arid steppe.

### Relationships among precipitation variability, plant productivity, and community

Integrating observations of global terrestrial ecosystem, we divided the collected results into different treatments (Griffin-Nolan et al., 2021), and their precipitation variability gradually decreased. By comparing the variable quantity across these treatments, the impacts of precipitation size and timing variability on global terrestrial ecosystems can be understood in general. In brief, in difference precipitation frequency, the timing uniformity of precipitation events had greater effects on plant productivity (**Figures 5A-C**), whereas the combination of increased precipitation event size and timing variability had greater effects on plant community structure (**Figures 5E-I**). In addition, in the same precipitation frequency group, all treatments slightly affected plant productivity and community structure (**Figures 5K-M**). Furthermore, we estimated the relationship among plant productivity and community and soil moisture in an altered precipitation pattern and found that BGB, diversity, and evenness were the most significantly affected by soil moisture change but were limited by linear description (**Table 1**; **Figures S2-S8**).

Our results showed that the timing uniformity of precipitation events had a greater effect on ANPP (**Figure 5C**), inconsistent with previously results, which indicated that variability in precipitation event size was reduced, timing increased ANPP, and increase in ANPP was correlated with increase in soil moisture and consecutive disparity in soil moisture, lower soil moisture variability (Griffin-Nolan et al., 2021). One reason for this difference may be difference in response between an ecosystem type and global terrestrial ecosystem. We then studied the relationship between terrestrial ecosystem and soil moisture affected by precipitation pattern, and found that ANPP was associated with high soil moisture variation, and had a quartic polynomial fitting relationship with the consecutive disparity index of soil moisture (**Table 1**; **Figure S2**). Studies have shown that the correlation between ANPP and precipitation presents an asymmetric response under the influence of spatial models, and the relationship between them is suitable for a nonlinear concave-down relationship when precipitation years are extreme (Knapp et al., 2017). Our results showed that the correlation between ANPP and soil moisture variability decreased gradually and exponentially in each group, although we failed to find an appropriate fitting function (**Table 1**; **Figures S2 and S5**). This finding indicated that the relationship between soil moisture change and ANPP is complicated in an altered precipitation pattern.

We found that the linear relationship was inadequate to describe variation in BGB with soil moisture (**Table 1**; **Figures S2 and S5**), and other studies have shown that underground production has greater stability in response to intra-annual precipitation variability (Griffin-Nolan et al., 2021), partially consistent with our results. Our results indicated that BGB had stronger resistance when soil water variability was small and the resistance of BGB decreased gradually with increasing soil moisture variability. This result suggested that subsurface processes play a major role responding to precipitation variability and thereby enable ecosystems to buffer this effect. In addition, we found that diversity and evenness first increased and then decreased with increasing soil moisture, and a study showed that the increase of precipitation variability promoted diversity, but we did not find this phenomenon (**Table 1**; **Figures S3 and S6**). Our results show that complex variations in precipitation pattern cause soil moisture variability alter, and the above and below ground productivity of plant is resistant when the variability is slight, but with the increase of soil moisture variability, the resistance effect of plant will gradually weaken, ultimately affecting the community composition of the ecosystem.

### Implications and future research

By conducting a meta-analysis, we quantitatively evaluated the patterns of the responses of terrestrial ecosystem structure and function to intra-annual precipitation pattern on a global scale. Our results indicated that terrestrial ecosystems are affected by altered intra-annual precipitation pattern (**Figure 2**), the timing uniformity of precipitation events is a critical factor for ecosystem functioning, and increase in precipitation variability is conducive to ecosystem community structure (**Figure 5**). Our results highlighted the importance of precipitation pattern to terrestrial ecosystems and implied the importance of considering the influence of variation in precipitation pattern on global carbon cycle apart from changes in precipitation amount.

Altered intra-annual precipitation pattern decreased ANPP and diversity but enhanced BNPP, richness, and relative abundance (**Figure 2**). However, few field experiments have been conducted on the effects of precipitation redistribution on terrestrial ecosystems when annual precipitation is constant. These limited data may cause inaccuracy in our conclusions. In addition, owing to the complexity of precipitation patterns, we failed to convert precipitation pattern into precipitation magnitude (Wang et al., 2021a) or divide it into increased or decreased precipitation (Wang et al., 2021b) to separately consider its impact on terrestrial ecosystem in the same manner as it was in other meta-analyses. Our current understanding of the impact of intra-annual precipitation pattern on ecosystem productivity across temporal and spatial scales is still limited. We considered the effects of ecosystem types on plant productivity and community structure (**Figure 3**; **Figure S1**). Most existing studies have been conducted in grassland ecosystems, and few experiments were conducted in peatland, savannas, and other ecosystems. However, owing to the complex processes and factors involved, different ecosystems may have different responses to precipitation patterns (Holmgren et al., 2013), and thus future studies need to consider the responses of other ecosystem types to precipitation pattern.

We assessed the impact of the size and timing variability of precipitation events on ecosystems (**Figure 5**). Given the precipitation times of the ambient and the treatment is a controlling factor, in the studies we collected, most of the precipitation times of ambient and treatment were distinct. Although eligible articles were few, a large proportion of data collected from the same literature. Therefore, such a small amount of data severely limited the assessment of precipitation event size and timing variability impact on global ecosystem structure and function. This limitation should be considered in future field rainfall experiments. An altered precipitation pattern may exert an impact on plant productivity by affecting soil moisture and nutrients (Yahdjian and Sala, 2010; Jones et al., 2016). Owing to the different monitoring methods and reported data of soil moisture dynamics in the articles we collected, uncertainty in our soil moisture data for assessing its impact on productivity may be present. In addition, the lack of soil nutrient data limited assessment of the impact of altered precipitation pattern. Therefore, the effects of altered precipitation patterns on soil nutrients should be considered in future works. Finally, the timing and size of a precipitation event are the two most important aspects that affect ecosystem structure and function. However, most current experiments have only used a single indicator of ecosystem function to evaluate the impact of precipitation pattern and rarely have paid attention to the response of community dynamics to intra-annual precipitation patterns. Thus, the importance of variability in precipitation timing and size in ecosystems cannot be fully assessed. Future studies should focus on the effects of variability in precipitation timing and size on community stability in ecosystems.

## Conclusions

Our study presents a general pattern of plant productivity and community structure, the dynamic response of soil moisture to altered precipitation patterns, and its relationship to plant productivity and community structure. BNPP increased in the total precipitation pattern, while ANPP decreased in different precipitation group, and species richness and relative abundance increased and diversity decreased under both groups. We also found that the timing uniformity of precipitation promoted the increase of ANPP, while the increase of precipitation variability was beneficial to plant community structure and the relationship between plant productivity and community structure and soil moisture was limited by a linear model. The results further elucidate the intrinsic link between plant productivity, community structure and precipitation pattern relationships, and plant productivity and soil moisture. The differences in plant productivity, community structure, and soil moisture indicate the importance of precipitation pattern as a driver of ecosystem processes for biome-specific.

## Data availability statement

The original contributions presented in the study are included in the article/**Supplementary Material**. Further inquiries can be directed to the corresponding author.

## Author contributions

MX analyzed data and wrote the manuscript. LL conceived the work and supervised this research. BL, YL and QW contributed to analyze of the manuscript. All authors contributed to the article and approved the submitted version.
