# Supplementary material for: Responses of terrestrial ecosystem productivity and community structure to intra-annual precipitation patterns: A meta-analysis
Source: Front Plant Sci. 2023 Jan 9;13:1088202. doi: 10.3389/fpls.2022.1088202 (PMC9868929; doi:10.3389/fpls.2022.1088202)
Supplement: Supplementary file 1 [file DataSheet_1.pdf]

Supporting Information

Responses of terrestrial ecosystem productivity and community structure to intra-annual precipitation patterns: A meta-analysis

Mingyu Xie [1,2,3,4], Lei Li [1,2,3,4], Bo Liu [5], Yalan Liu [1,2,3,4] and Qian Wan [1,2,3,4]*

[1] State Key Laboratory of Desert and Oasis Ecology, Xinjiang Institute of Ecology and Geography, Chinese Academy of Sciences, Urumqi, China, [2] Xinjiang Key Laboratory of Desert Plant Roots Ecology and Vegetation Restoration, Xinjiang Institute of Ecology and Geography, Chinese Academy of Sciences, Urumqi, China, [3] Cele National Station of Observation and Research for Desert-Grassland Ecosystems, Cele, China, [4] University of Chinese Academy of Sciences, Beijing, China, [5] Shandong Provincial Key Laboratory of Soil Conservation and Environmental Protection, College of Resources and Environment, Linyi University, Linyi, China

Correspondence: Lei Li lilei@ms.xjb.ac.cn

Contents of this file

1. TABLE S2 | Response variables used in this meta-analysis

2. TABLE S3 | Between-group heterogeneity test (Q_B) for ecosystem types, precipitation distribution and periods

3. FIGURE S1 | Subgroup analysis of productivity and community structure indicators on altered precipitation pattern distribution.

4. FIGURE S2 | The linear and nonlinear relationships between plant productivity and soil moisture in total precipitation pattern.

5. FIGURE S3 | Responses of plant community structure indices to soil moisture in total precipitation pattern.

6. FIGURE S4 | The relationships of plant community structure with soil moisture indices in total precipitation pattern.

7. FIGURE S5 | Regression analysis of plant productivity and soil moisture under different precipitation frequency.

8. FIGURE S6 | Responses of plant community structure to soil moisture indices in different precipitation pattern.

9. FIGURE S7 | The relationships of plant community structure with soil moisture indices in different precipitation pattern.

10. FIGURE S8 | Responses of plant productivity and community structure indices to soil moisture in same precipitation frequency.

TABLE S2 | Response variables used in this meta-analysis

Response variables	Definition
**Biomass**	
Aboveground biomass (AGB, g m^{-2})	All living aboveground dry mass per unit area
Belowground biomass (BGB, g m^{-2})	All living dry mass of living roots per unit area
**Net primary productivity**	
Aboveground net primary productivity (ANPP, g m^{-2} yr^{-1})	Dry mass of all living aboveground growth per unit area per unit time
Belowground net primary productivity (BNPP, g m^{-2} yr^{-1})	Dry mass of root growth per unit area per unit time
**Community structure indicators**	
Species richness	The number of species per unit of area
Cover (%)	The percentage of the vertical projection of vegetation (including leaves, stems, and branches) on the ground to the total area of a particular area
Shannon-Wiener (*H*)	Reflects the diversity of plant community based on the number of species
Pielou's evenness index (*E*)	The degree to which abundance (biomass, coverage, or other index) of different species is distributed evenly in a community
Relative abundance (%)	The abundance of one species as a percentage of the total abundance of all species in a community
**Root-shoot ratio (R/S)**	The ratio of fresh or dry weight between belowground and aboveground parts of a plant

TABLE S3 | Between-group heterogeneity test (Q_B) for ecosystem types, precipitation distribution and periods

Variables	Ecosystem types		Same frequency		Different frequency		Periods	
	Q_B	*p*-value	Q_B	*p*-value	Q_B	*p*-value	Q_B	*p*-value
AGB	14.311	0.000***	NA	NA	13.764	0.000***	0.791	0.374
BGB	14.336	0.000***	0.845	0.655	9.879	0.002**	0.004	0.948
ANPP	53.781	0.000***	NA	NA	42.293	0.000***	23.523	0.000***
BNPP	0.265	0.607	NA	NA	NA	NA	0.265	0.607
SR	6.005	0.049*	NA	NA	0.750	0.387	NA	NA
E	NA	NA	NA	NA	NA	NA	1.859	0.173
RA	5.521	0.063	NA	NA	5.521	0.063	0.056	0.814
D	0.185	0.667	NA	NA	0.185	0.667	0.450	0.502
R/S	11.153	0.004**	NA	NA	11.153	0.004**	11.272	0.000***
C	3.507	0.320	0.410	0.938	13.486	0.000***	0.229	0.632

p-values < 0.05 are considered significant

*$p < 0.05$ **$p < 0.01$ *** $p < 0.001$

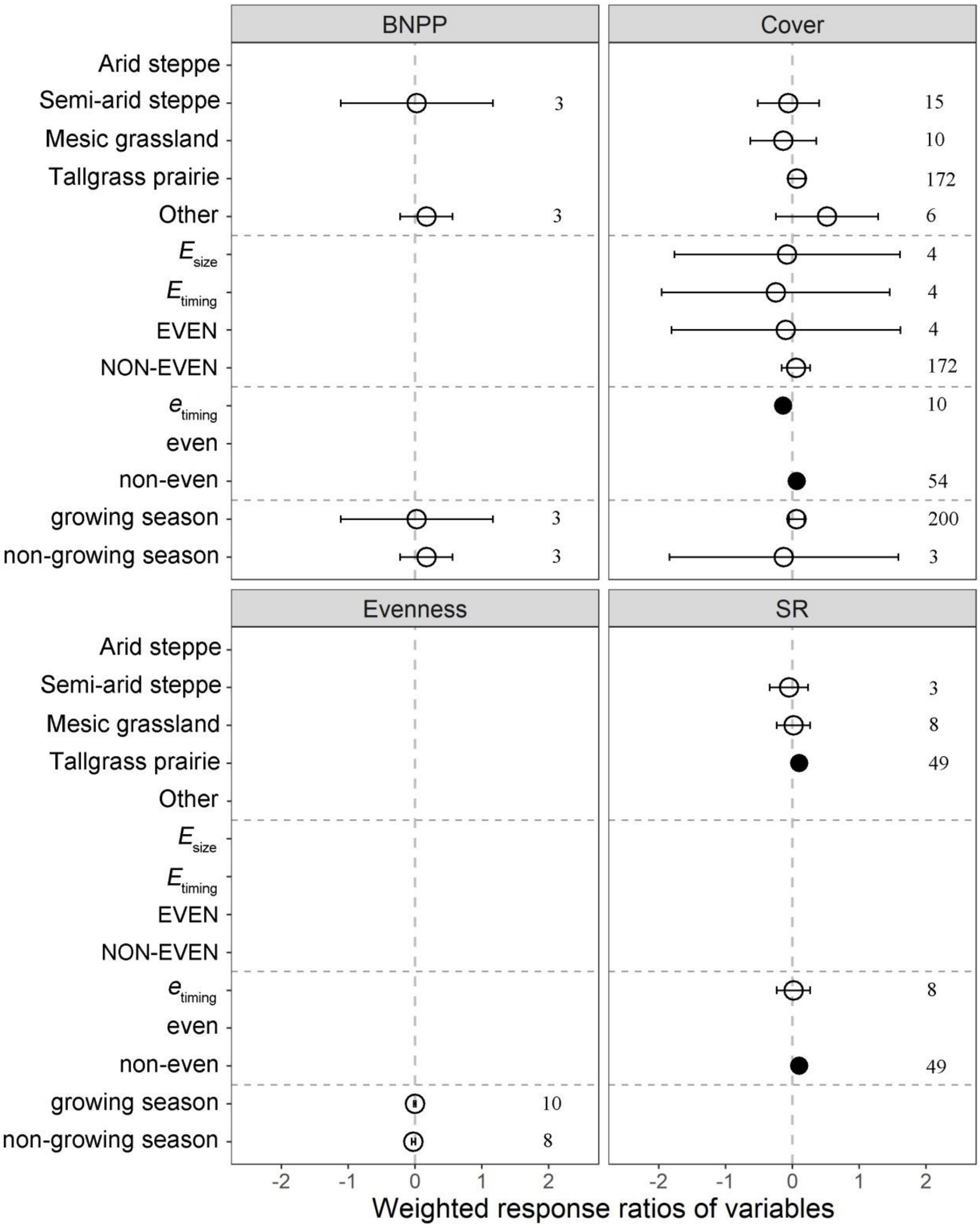

FIGURE S1 | Subgroup analysis of plant productivity and community structure on altered intra-annual precipitation patterns. Between-group heterogeneity (Q_B) was tested according to ecosystem types, precipitation distribution and experimental period. If 95% CIs did not overlap with zero, the effects of precipitation pattern on variables were considered significant (denoted by black circles). A significant Q_B value ($p < 0.05$) suggested that the weighted response ratios of a given variable differed among groups. Numbers indicate the number of data observations. Error bars represent 95% confidence intervals (CI). The vertical dashed line represents weighted response ratios = 0. See **Figure 2** and **Figure 3** for abbreviations.

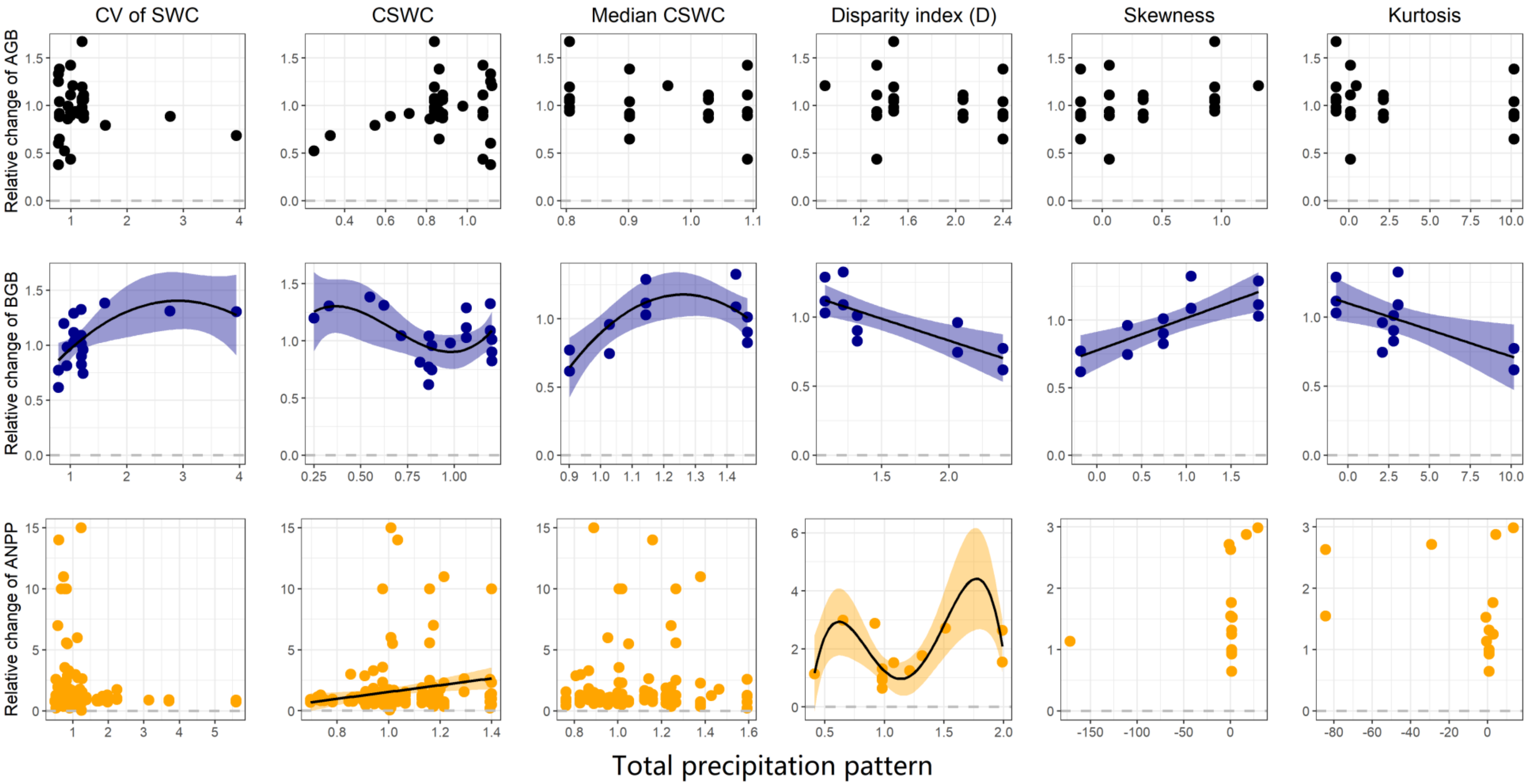

FIGURE S2 | The linear and nonlinear relationships between plant productivity and soil moisture in total precipitation pattern. The shaded region represents the 95% confidence interval for the relationship. The implications of soil moisture indices are defined in the legend to **Figure 4**.

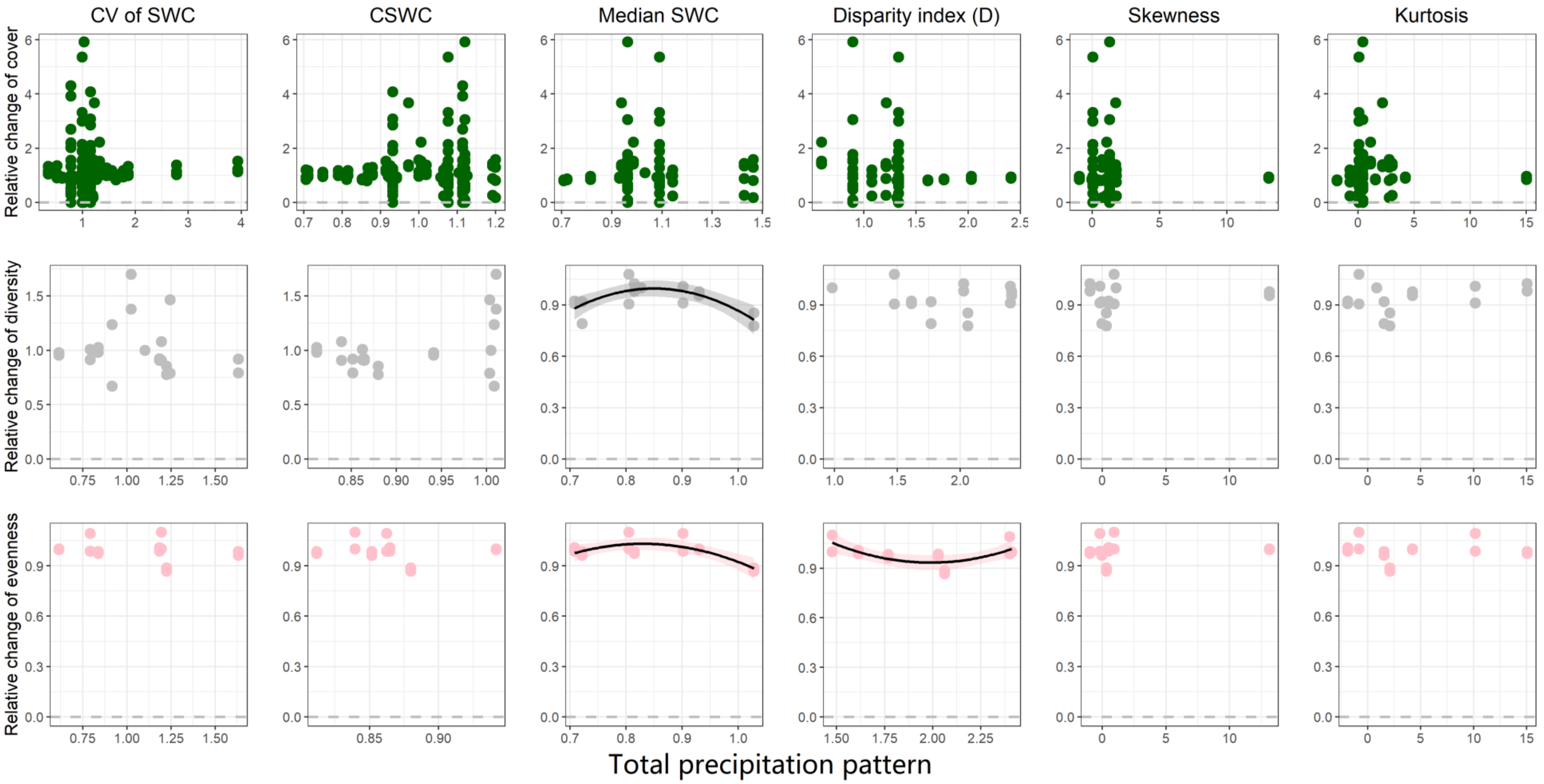

FIGURE S3 | Responses of plant community structure indices to soil moisture in total precipitation pattern. Nonlinear regressions and their 95% confidence intervals (shaded areas) are presented. The implications of soil moisture indices are defined in the legend to **Figure 4**.

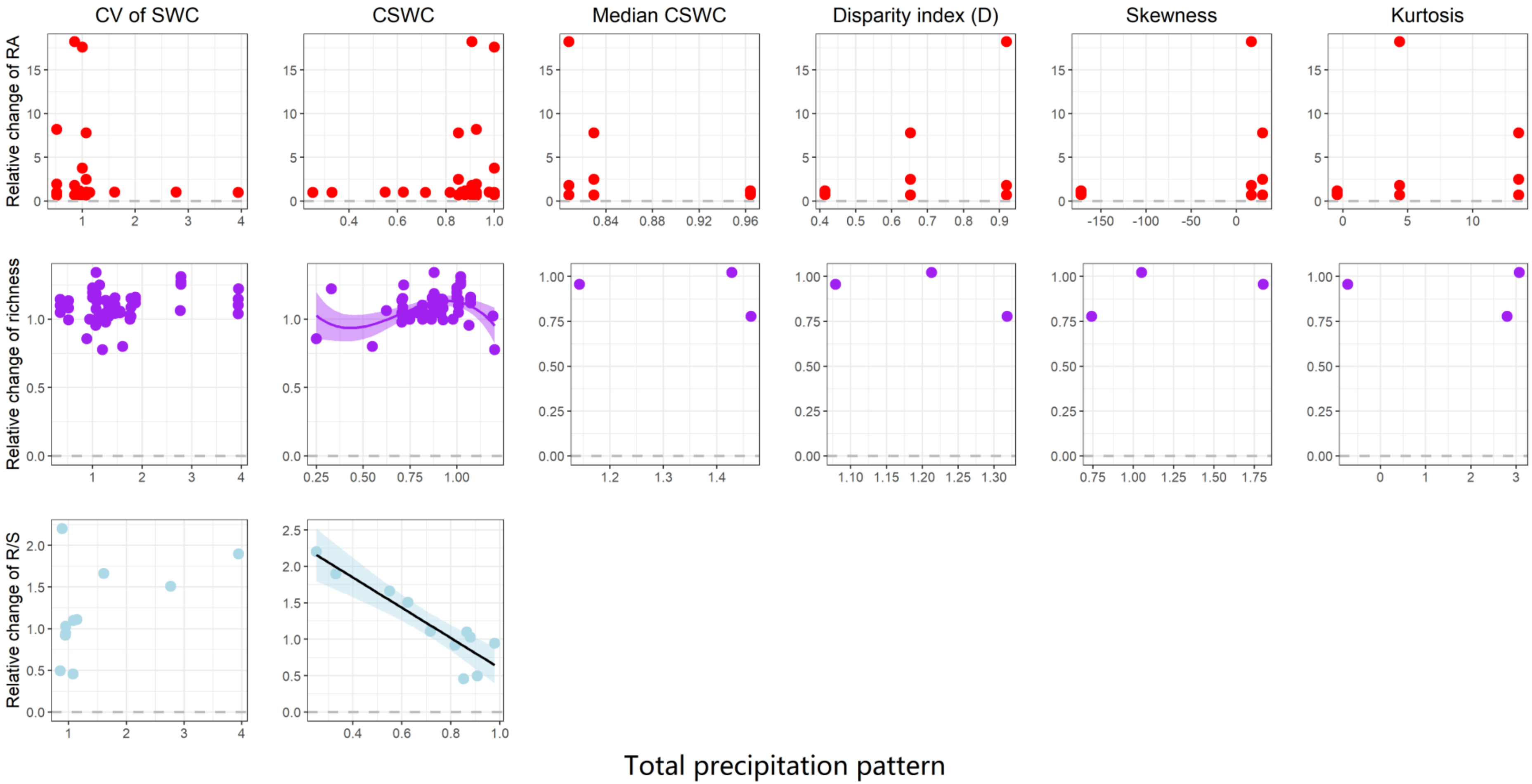

FIGURE S4 | The relationships of plant community structure with soil moisture indices in total precipitation pattern. The shaded region represents the 95% confidence interval for the linear and nonlinear regressions. The implications of soil moisture indices are defined in the legend to **Figure 4.**

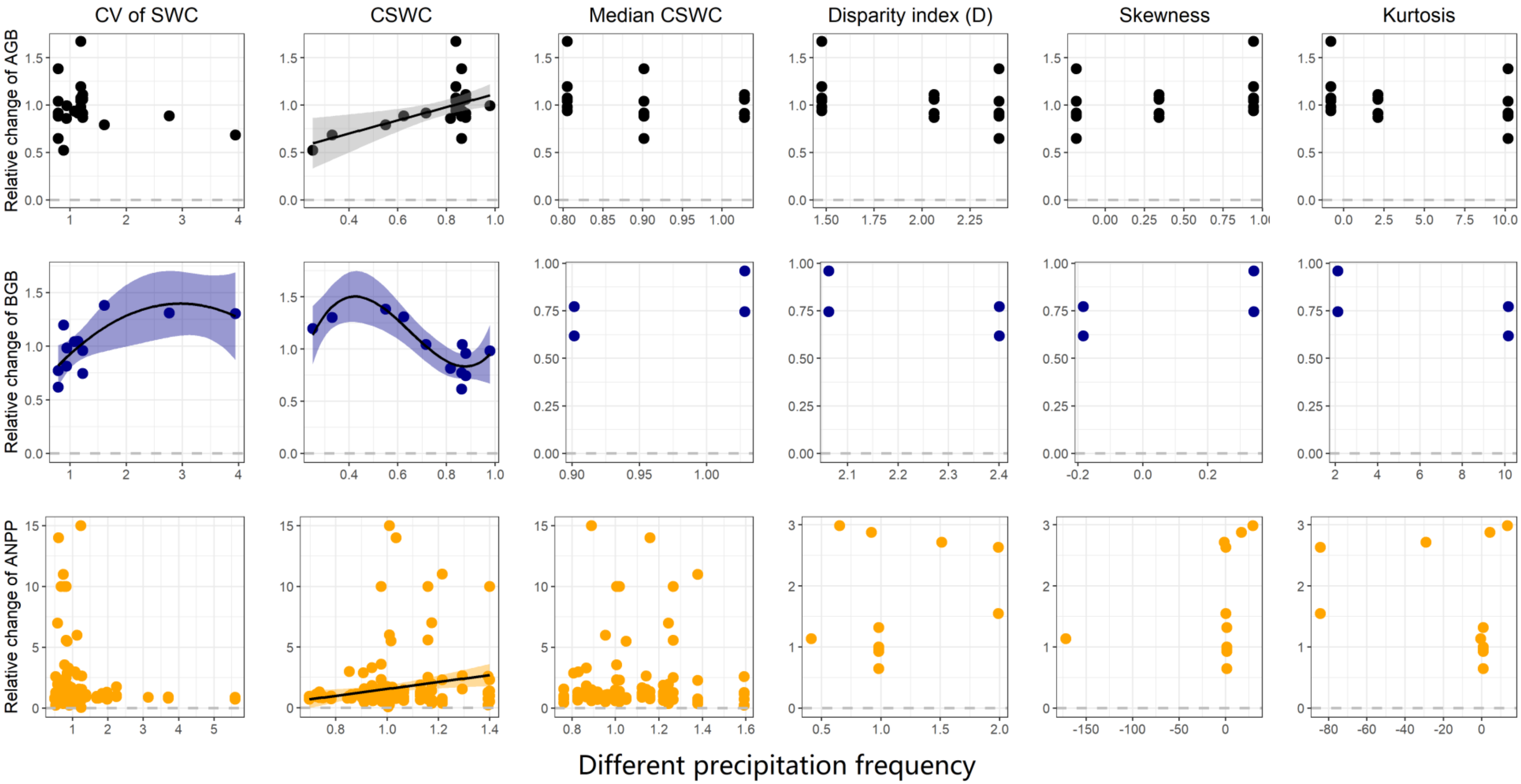

FIGURE S5 | Regression analysis of plant productivity and soil moisture under different precipitation frequency. The shaded region represents the 95% confidence interval for the relationship. The implications of soil moisture indices are defined in the legend to **Figure 4**.

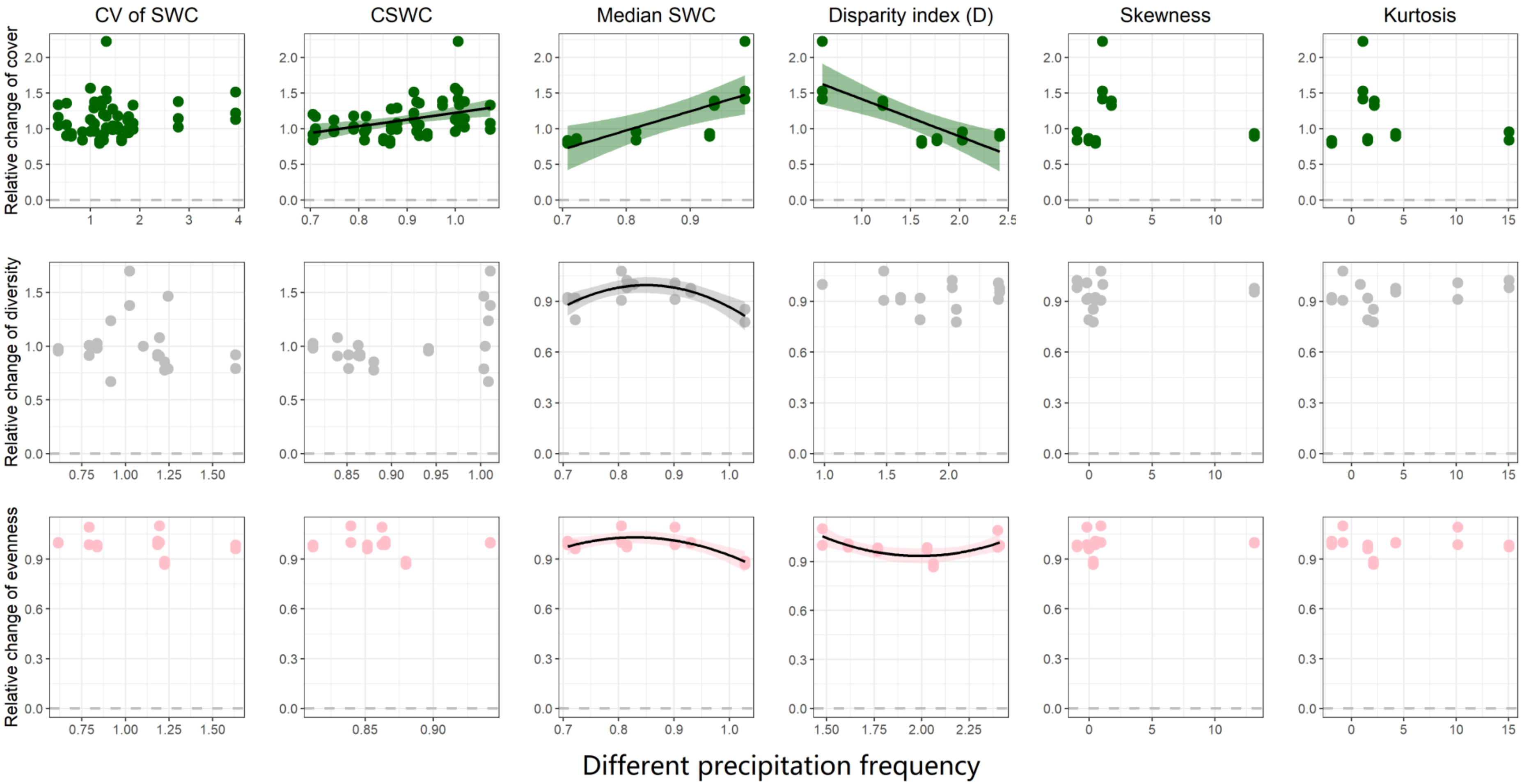

FIGURE S6 | Responses of plant community structure to soil moisture indices in different precipitation pattern. Nonlinear regressions and their 95% confidence intervals (shaded areas) are presented. The implications of soil moisture indices are defined in the legend to **Figure 4**.

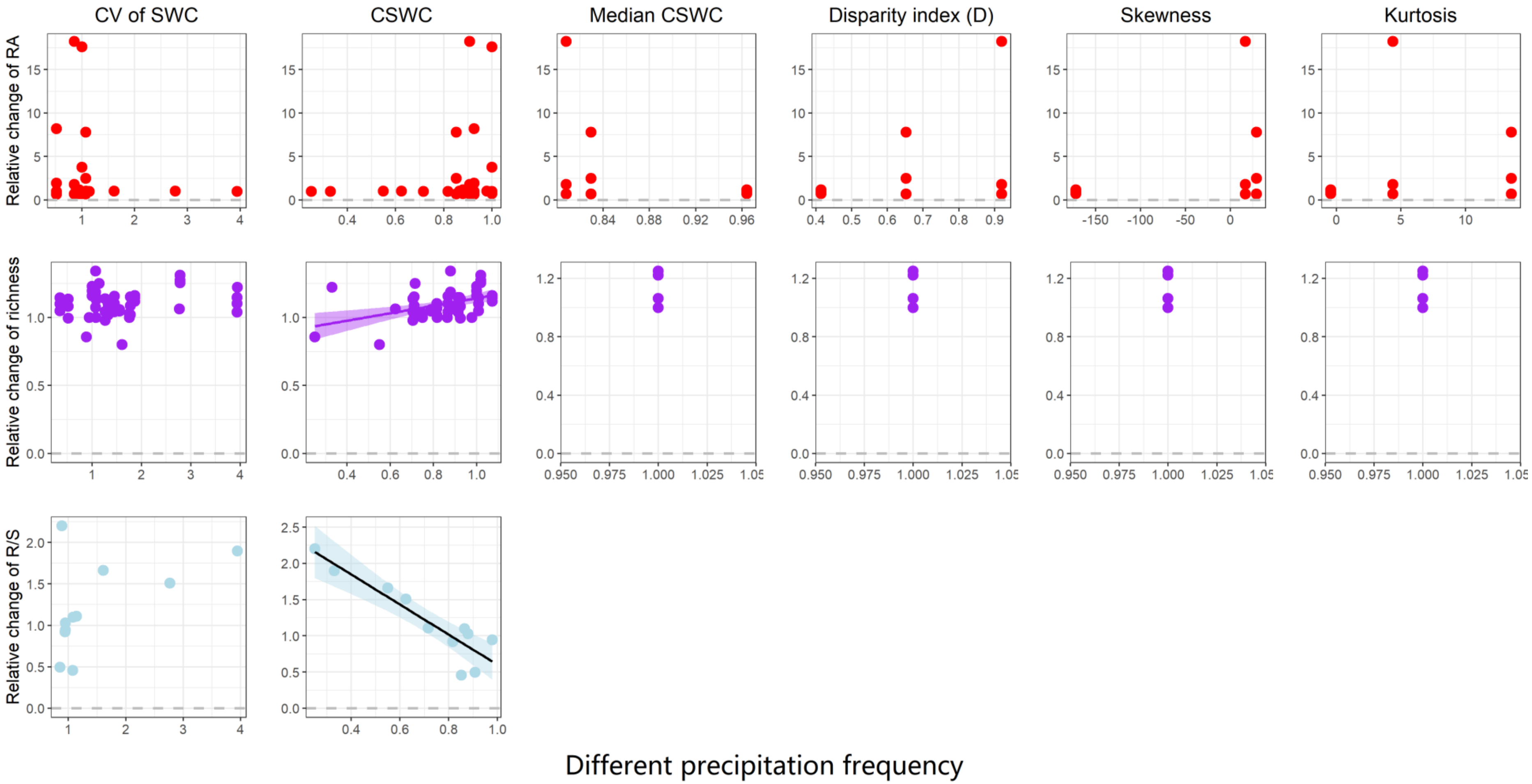

FIGURE S7 | The relationships of plant community structure with soil moisture indices in different precipitation pattern. The shaded region represents the 95% confidence interval for the linear and nonlinear regressions. The implications of soil moisture indices are defined in the legend to **Figure 4**.

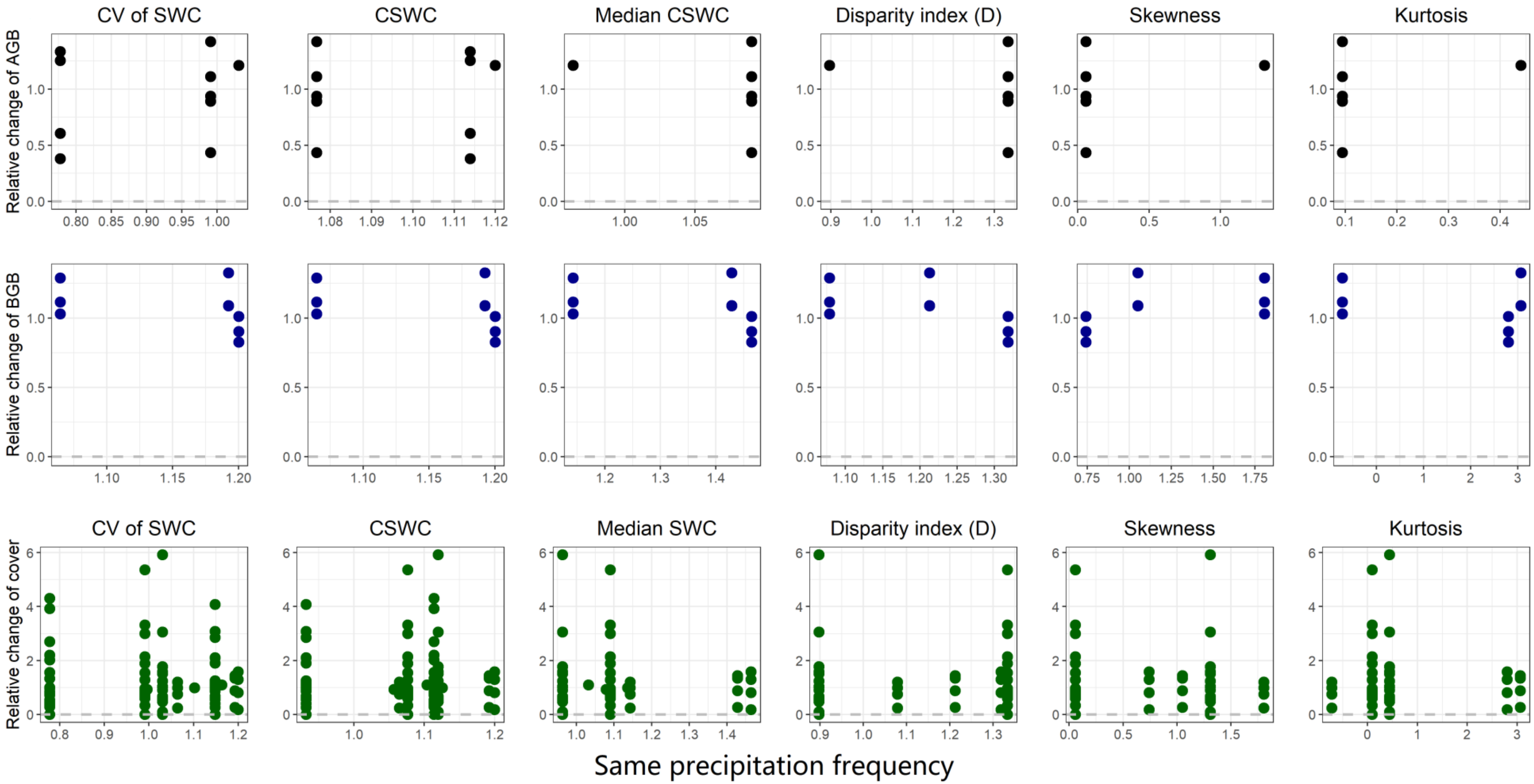

FIGURE S8 | Responses of plant productivity and community structure indices to soil moisture in same precipitation frequency. The implications of soil moisture indices are defined in the legend to **Figure 4**.
