# Supplementary material for: Responses of terrestrial ecosystem productivity and community structure to intra-annual precipitation patterns: A meta-analysis
Source: Front Plant Sci. 2023 Jan 9;13:1088202. doi: 10.3389/fpls.2022.1088202 (PMC9868929; doi:10.3389/fpls.2022.1088202)
Supplement: Supplementary file 2 [file DataSheet_2.docx]

**Supplementary File 1 – Data sources**

Avolio, M. L., Beaulieu, J. M., & Smith, M. D. (2013). Genetic diversity of a dominant C_4_ grass is altered with increased precipitation variability. *Oecologia, 171*, 571-581. <https://doi.org/10.1007/s00442-012-2427-4>

Barnett, K. L., Johnson, S. N., Facey, S. L., Gibson-Forty, E. V. J., Ochoa-Hueso, R., & Power, S. A. (2021). Altered precipitation and root herbivory affect the productivity and composition of a mesic grassland. *BMC Ecology and Evolution, 21*. <https://doi.org/10.1186/s12862-021-01871-0>

Fay, P. A., Blair, J. M., Smith, M. D., Nippert, J. B., Carlisle, J. D., & Knapp, A. K. (2011). Relative effects of precipitation variability and warming on tallgrass prairie ecosystem function. *Biogeosciences, 8*, 3053-3068. <https://doi.org/10.5194/bg-8-3053-2011>

Griffin-Nolan, R. J., Slette, I. J., & Knapp, A. K. (2021). Deconstructing precipitation variability: Rainfall event size and timing uniquely alter ecosystem dynamics. *Journal of Ecology, 109*, 3356-3369. <https://doi.org/10.1111/1365-2745.13724>

Heisler-White, J. L., Blair, J. M., Kelly, E. F., Harmoney, K., & Knapp, A. K. (2009). Contingent productivity responses to more extreme rainfall regimes across a grassland biome. *Global Change Biology, 15*, 2894-2904. <https://doi.org/10.1111/j.1365-2486.2009.01961.x>

Jones, S. K., Collins, S. L., Blair, J. M., Smith, M. D., & Knapp, A. K. (2016). Altered rainfall patterns increase forb abundance and richness in native tallgrass prairie. *Scientific Reports, 6*. <https://doi.org/10.1038/srep20120>

Jongen, M., Lecomte, X., Unger, S., Pinto-Marijuan, M., & Pereira, J. S. (2013). The impact of changes in the timing of precipitation on the herbaceous understorey of Mediterranean evergreen oak woodlands. *Agricultural and Forest Meteorology, 171*, 163-173. <https://doi.org/10.1016/j.agrformet.2012.11.020>

Jongen, M., Unger, S., Fangueiro, D., Cerasoli, S., Silva, J. M. N., & Pereira, J. S. (2013). Resilience of montado understorey to experimental precipitation variability fails under severe natural drought. *Agriculture Ecosystems & Environment, 178*, 18-30. <https://doi.org/10.1016/j.agee.2013.06.014>

Koerner, S. E., Collins, S. L., Blair, J. M., Knapp, A. K., & Smith, M. D. (2014). Rainfall variability has minimal effects on grassland recovery from repeated grazing. *Journal of Vegetation Science, 25*, 36-44. <https://doi.org/10.1111/jvs.12065>

Liu, W. J., Li, L. F., Biederman, J. A., Hao, Y. B., Zhang, H., Kang, X. M., . . . Xu, C. Y. (2017). Repackaging precipitation into fewer, larger storms reduces ecosystem exchanges of CO_2_ and H_2_O in a semiarid steppe. *Agricultural and Forest Meteorology, 247*, 356-364. <https://doi.org/10.1016/j.agrformet.2017.08.029>

Meng, B., Li, J., Maurer, G. E., Zhong, S., Yao, Y., Yang, X., . . . Sun, W. (2021). Nitrogen addition amplifies the nonlinear drought response of grassland productivity to extended growing-season droughts. *Ecology, 102*, e03483. <https://doi.org/10.1002/ecy.3483>

Radu, D. D., & Duval, T. P. (2018). Precipitation frequency alters peatland ecosystem structure and CO_2_ exchange: Contrasting effects on moss, sedge, and shrub communities. *Global Change Biology, 24*, 2051-2065. <https://doi.org/10.1111/gcb.14057>

Ru, J., Zhou, Y., Hui, D., Zheng, M., & Wan, S. (2017). Shifts of growing-season precipitation peaks decrease soil respiration in a semiarid grassland. *Glob Chang Biol, 24*, 1001-1011. <https://doi.org/10.1111/gcb.13941>

Schuster, M. J., Smith, N. G., & Dukes, J. S. (2016). Responses of aboveground C and N pools to rainfall variability and nitrogen deposition are mediated by seasonal precipitation and plant community dynamics. *Biogeochemistry, 129*, 389-400. <https://doi.org/10.1007/s10533-016-0240-6>

Smith, N. G., Schuster, M. J., & Dukes, J. S. (2016). 18 Rainfall variability and nitrogen addition synergistically reduce plant diversity in a restored tallgrass prairie. *Journal of Applied Ecology, 53*, 579-586. <https://doi.org/10.1111/1365-2664.12593>

Zhang, Z., Shan, L., & Li, Y. (2017). Prolonged dry periods between rainfall events shorten the growth period of the resurrection plant Reaumuria soongorica. *Ecology and Evolution, 8*, 920-927. <https://doi.org/10.1002/ece3.3614>
